# Non-Invasive Jaundice Screening Using AI: Machine Learning Analysis of Sclera and Urine Images

**DOI:** 10.3390/jcm14093125

**Published:** 2025-04-30

**Authors:** Jungirl Seok, Jae Yeong Kim, Sang Gyune Kim, Young Seok Kim, Jeong-Ju Yoo

**Affiliations:** 1 Department of Otorhinolaryngology-Head and Neck Surgery, Seoul National University Hospital, Seoul National University College of Medicine, Seoul 03080, Republic of Korea; junn279@gmail.com (J.S.); rlawodud112294@gmail.com (J.Y.K.); 2 Department of Otorhinolaryngology-Head and Neck Surgery, National Cancer Center, Goyang-si 10408, Republic of Korea; 3 Division of Gastroenterology and Hepatology, Department of Internal Medicine, Soonchunhyang University Bucheon Hospital, Bucheon 14584, Republic of Korea; mcnulty@schmc.ac.kr (S.G.K.); liverkys@schmc.ac.kr (Y.S.K.)

**Keywords:** non-invasive jaundice prediction, machine learning, sclera and urine image analysis, bilirubin level detection

## Abstract

**Background:** Jaundice commonly indicates liver dysfunction and is traditionally diagnosed via invasive blood tests. **Objective:** This study aimed to develop an AI-based program utilizing images of sclera and urine for non-invasive jaundice screening and compared its accuracy to that of standard blood tests. **Methods**: This retrospective study involved patients who underwent liver function and bilirubin tests. Scleral and urine images were collected and processed following a standardized protocol to ensure consistency; A4 paper was utilized for white balance correction. Various machine learning and deep learning algorithms, including Decision Tree, Random Forest, XGBoost, DeepSets, and ResNet, were applied to predict jaundice. A stratified five-fold cross-validation was employed, and jaundice was classified as present or absent based on the total bilirubin levels. **Results:** In total, 57 patients with liver disease and 31 controls were included in the analysis. Various machine learning models were applied to analyze scleral and urine images. The DeepSets model exhibited the highest predictive performance, achieving an R^2^ of 0.782 in predicting bilirubin levels. For jaundice detection, the DeepSets model, using a threshold of 2.6 mg/dL, achieved an accuracy of 87.1% (AUC = 0.869), with a precision of 90.2% and a recall of 88.1%. In contrast, the Random Forest model, with a threshold of 2.9 mg/dL, achieved an accuracy of 84.2% (AUC = 0.833), with a precision of 86.0%, and a recall of 88.1%. **Conclusions:** This study demonstrates that scleral images, when used with simple A4 white paper for color correction, have the potential to screen for jaundice and predict bilirubin levels. (Clinical Research Information Service of Republic of Korea, KCT0009915).

## 1. Introduction

Jaundice is a common clinical condition characterized by the yellowing of the skin and sclera, which results from elevated bilirubin levels in the blood. This condition may signal various underlying health issues, such as liver disease, hemolysis, or bile duct obstruction [1]. The early detection and accurate diagnosis of jaundice are critical for the effective treatment and management of these underlying conditions [2,3].

Non-invasive screening techniques have several advantages; they reduce discomfort and anxiety for patients, lower the risk of infection, and are more accessible, especially in resource-limited settings [3,4]. In addition, non-invasive techniques enable faster clinical decision-making and early interventions. The ability to perform rapid and frequent screenings can improve patient outcomes and reduce healthcare costs [5]. In particular, bilirubin levels are traditionally measured through blood tests. However, there is no dedicated screening test specifically for jaundice.

Recent advancements in medical technology have underscored the potential of non-invasive diagnostic techniques. Clinically, evaluating jaundice starts with distinguishing unconjugated from conjugated hyperbilirubinemia. Unconjugated forms relate to increased production (e.g., hemolysis), impaired uptake, or defective conjugation (e.g., Gilbert’s syndrome). Conjugated forms are due to hepatocellular injury or cholestasis—either intrahepatic (e.g., hepatitis, drugs, sepsis) or extrahepatic (e.g., bile duct obstruction). This biochemical approach and classification help narrow down causes and further guide workup. The yellow discoloration of the sclera and changes in urine color are hallmark symptoms of jaundice, presenting opportunities for the development of non-invasive diagnostic tools [2,6,7]. Dark urine is often an early sign of conjugated hyperbilirubinemia, reflecting the increased renal excretion of water-soluble bilirubin. It commonly occurs in the setting of hepatocellular injury or cholestasis and may appear before overt jaundice becomes clinically evident. However, dark urine is not specific to liver disease and can also result from hematuria, hemoglobinuria, or myoglobinuria. Therefore, a brief visual assessment of urine color may serve as a simple screening clue for hepatic dysfunction when interpreted in the proper clinical context.

Leveraging recent advances in machine learning and artificial intelligence, combined with image processing techniques, holds significant clinical promise. These technologies can analyze visual data with high precision, potentially identifying jaundice symptoms with greater accuracy and speed than traditional methods. In this study, we aimed to develop an artificial intelligence program capable of predicting jaundice based on sclera images and changes in urine color. Our objective was to assess whether this program can serve as an effective screening tool in clinical settings and to compare its accuracy with that of the gold standard—namely, blood tests. This paper presents the development process, technical specifications, and validation results of our program, illustrating its potential for jaundice screening based on experimental findings.

## 2. Materials and Methods

The Transparent Reporting of an artificial intelligence (AI)-powered multivariable prediction model for Individual Prognosis Or Diagnosis (TRIPOD + AI) checklist is provided in Supplementary Table S1 [8]. All authors had access to the study data and reviewed and approved the final manuscript.

### 2.1. Ethical Considerations

The study protocol was reviewed and approved by the Institutional Review Board of the Soonchunhyang University College of Medicine (IRB No. SCHBC 2023-12-009-001). The study was registered on cris.nih.go.kr (Identifier: Clinical Research Information Service (CRiS) of Republic of Korea, KCT0009915). Informed consent was waived due to the retrospective design of the study.

### 2.2. Patients and Acquisition of Pictures of Sclera and Urine

The study included patients who visited the gastroenterology department with abnormal liver function, liver disease, or biliary tract disease and underwent blood and urine tests from October 2022 to October 2023. As a control group, we enrolled patients who visited the same center but had no specific underlying liver or biliary tract disease and were at low risk for jaundice and hyperbilirubinemia.

**Figure 1:**
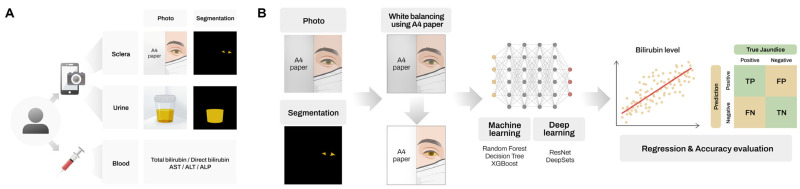
Schematic overview of the study design. Scleral and urine photographs and blood test results were obtained from patients (**A**). Representative example of the AI pipeline illustrated using scleral image analysis (**B**) Overview of the AI pipeline for predicting bilirubin levels using scleral images.

Scleral photography was performed by placing a mask over the patient’s nose and mouth, exposing only one eye. Each patient had only one scleral photograph taken from the right eye. Consequently, each patient’s scleral image was used only once, either in the training or validation set. An A4 sheet from Hankuk Paper (Ulsan, Republic of Korea) was positioned beside the patient’s face, occupying half of the screen. The inclusion of the A4 paper in the photograph aimed to correct the white balance, thus minimizing errors in scleral color between the different photographs (Figure 1).

Urine color was documented by placing the specimen container against a white background at the reception desk. All urine photographs were taken at this same fixed location under consistent lighting conditions, using the white surface of the background itself as a reference for white balancing.

All patient specimens were photographed at this same location. The photographs were captured using an iPhone 12 Pro (Apple Inc., Cupertino, CA, USA) with the live photo feature disabled and all other settings at their default values. The entire process, including explaining the procedure to the patient and completing the photograph acquisition, was completed within five minutes.

The study investigated various factors, including patients’ age, sex, underlying liver disease, total bilirubin, direct bilirubin, aspartate aminotransferase (AST), alanine transaminase (ALT), and alkaline phosphatase (ALP). The normal reference ranges at our institution were as follows: total bilirubin, 0.2–1.5 mg/dL; direct bilirubin, 0.0–0.2 mg/dL; AST, 5–45 U/L; ALT, 0–40 U/L; and ALP, 30–120 U/L. Blood tests and photography were conducted within a maximum time difference of three days.

A similar study with a comparable experimental design involving 130 patients reported a Pearson correlation coefficient of 0.7 (*p*-value < 0.001) between bilirubin levels and the measured parameters [9]. Based on this correlation, we estimated the minimum required sample size using Cohen’s effect size calculation for correlation studies. With a significance level (α) of 0.05 and a statistical power of 0.8, the required sample size was determined to be 17 patients. However, to enhance the robustness and generalizability of our findings, we collected a substantially larger sample size than this minimum requirement.

### 2.3. Primary Outcome and Evaluation

The primary outcome of this study was to predict bilirubin levels and the presence of jaundice using machine learning algorithms. For the prediction of bilirubin levels, we performed regression analysis to obtain the R^2^ value and *p*-value, assessing the strength and significance of the model’s predictions. For the diagnosis of jaundice, we used a confusion matrix to calculate key performance metrics, including accuracy, precision, recall, and the F1 score, to evaluate the classification capability of the model.

$$Accuracy=\frac{TP+TN}{TP+TN+FP+FN}$$

$$Precision=\frac{TP}{TP+FP}$$

$$Recall=\frac{TP}{TP+FN}$$

$$F1\;score=2\cdot \frac{Precision\cdot Recall}{Precision+Recall}$$

### 2.4. Definition of Threshold Values

The term “jaundice” is synonymous with hyperbilirubinemia, where normal serum levels of total bilirubin are typically less than 1 mg/dL [10]. Clinically, jaundice manifests as scleral icterus when serum bilirubin levels exceed 3 mg/dL [10]. Based on this clinical observation, we categorized bilirubin levels above 3 mg/dL as abnormal and those at or below this threshold as normal. Initially, we calculated the accuracy of the algorithm using this classification. After identifying the most effective algorithm, we then fine-tuned the bilirubin threshold used to differentiate between normal and abnormal cases. This threshold was adjusted from 1.0 to 4.0 in 0.1 mg/dL increments to establish the optimal level.

### 2.5. Image Analysis

The default settings were used for smartphone photography; however, this may lead to white balance issues, making it difficult to compare colors under the same conditions. To account for minor environmental variations, such as changes in lighting or the patient’s skin color, we used A4 paper to correct the colors. To enhance robustness against varying lighting conditions, we applied the von Kries transformation as follows:

$$D=D_{1}^{-1}D_{2}=\left[\begin{array}{ccc} \frac{L_{2}}{L_{1}} & 0 & 0\\ 0 & \frac{M_{2}}{M_{1}} & 0\\ 0 & 0 & \frac{S_{2}}{S_{1}} \end{array}\right]$$

**Figure 2:**
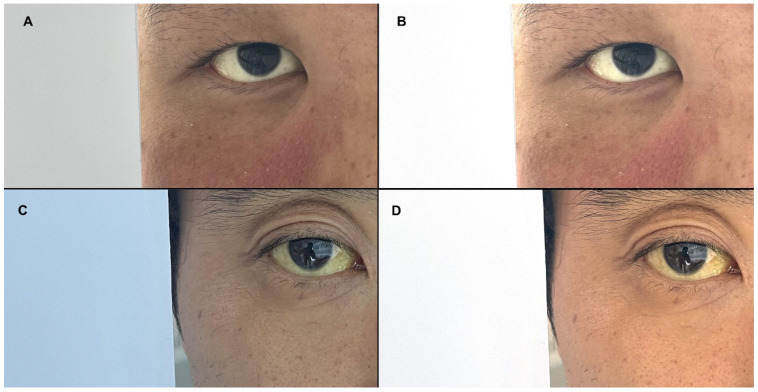
Representative images corrected using the von Kries transformation. Pre-correction (**A**,**C**) and post-correction images (**B**,**D**).

We used this formula to normalize the color and brightness of all images based on the pixel values of the white reference paper (Figure 2). The images were then converted to the YCbCr color space and analyzed [11].

Since bilirubin levels and the presence of jaundice directly influence the color of a patient’s sclera and urine, we developed a model to predict these factors based on pixel values from these regions. Although the latest deep learning techniques employ depth and complexity in their architecture, which could potentially eliminate the need for image correction, they are significantly limited by their requirement for large datasets. Given the limited dataset available for this study, we concentrated on extracting color data from specific regions of interest through image preprocessing, which is a process analogous to feature selection in traditional machine learning techniques. As a result, we implemented well-known conventional machine learning algorithms, such as Decision Tree, Random Forest, and XGBoost [12,13,14]. Additionally, we utilized the DeepSets model within a neural network framework [15]. Finally, we included the ResNet-18 and ResNet-50 models [16], which are fundamental deep learning algorithms in the convolutional neural network family, to compare overall performance. Among the ResNet models, we adopted ImageNet-pretrained weights, which are well-suited for achieving strong performance even with a smaller dataset [17].

The input structure (or input layer dimension) varied across the models. For ResNet-18 and ResNet-50, which are based on the CNN architecture, input images were resized to 224 × 224 × 3. For the DeepSets model, input data consisted of YCbCr values from the region of interest (RoI), which was determined by masking (width × height × 3). For conventional machine learning models, input was composed of a one-dimensional vector of 1567 extracted features. Further details are provided in Supplementary Text S1.

All experiments were conducted using stratified 5-fold cross-validation. To minimize the impact of randomness and reduce discrepancies between the distributions of the train and validation sets, we divided these sets based on total bilirubin levels. Initially, cases were categorized as normal or abnormal according to their bilirubin levels, and the sets were assembled to maintain a consistent ratio between these two groups. Further information on the fine-tuning of hyperparameters and the training settings for each algorithm is available in Supplementary Text S2.

### 2.6. Statistical Analysis

All statistical analyses were performed using Python (v3.9) and the SciPy and Scikit-learn libraries. Continuous variables were expressed as means ± standard deviations or medians with interquartile ranges (IQRs), depending on the distribution. Categorical variables were presented as frequencies and percentages. To compare the differences between the liver disease group and control group, we used the Wilcoxon rank-sum test for continuous variables, as the data were not normally distributed, and the Chi-square test for categorical variables. A *p*-value < 0.05 was considered statistically significant.

## 3. Results

In total, 57 patients with liver disease and 31 control subjects were included in the study (Table 1). There were no significant differences in age or sex between the two groups. In the liver disease group, the levels of total bilirubin and direct bilirubin were significantly higher than in the control group (both *p*-values < 0.001; Wilcoxon rank-sum test). Significant differences were also observed in AST and ALP levels (*p*-values < 0.001 and 0.003, respectively; both Wilcoxon rank-sum test). However, ALT levels did not differ significantly (*p*-value = 0.701). Among the patients, the causes of liver disease were as follows: alcoholic liver disease in 32 patients (56.1%), hepatitis B virus in 15 patients (26.3%), hepatitis C virus in 1 patient (1.8%), and other causes in 9 patients (15.8%).

### 3.1. Predicting Bilirubin Levels Using Various Algorithms

We applied six different algorithms to two types of data, scleral images, and urine images, along with an ensemble method that utilized both. The results indicated that using only scleral images yielded higher performance than the other two approaches, with the threshold set at 3.0 mg/dL (refer to Table 2 for scleral images and Supplementary Table S2 for urine images and the ensemble analysis). Both urine images and the ensemble approach resulted in a significant decline in performance across all models compared to the use of scleral images only. Notably, performance dropped most significantly when using only urine, with R^2^ values falling below 0.2 across all models. Consequently, further analysis was conducted exclusively on scleral images.

An example of white-balanced scleral photographs and the comparison between predicted and ground-truth bilirubin levels is provided in Supplementary Figure S1.

**Figure 3:**
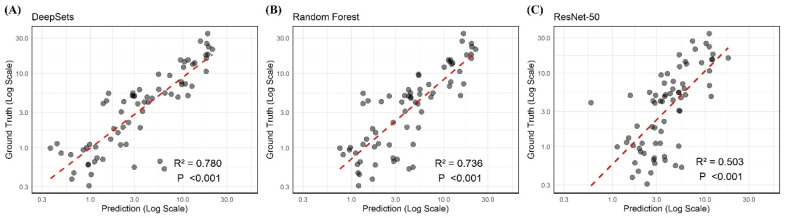
The regression results of DeepSets, Random Forest, and ResNet-50 using scleral images showing the two highest and lowest R^2^ values (0.780, 0.736, and 0.503), respectively.

All six algorithms demonstrated statistically significant results in regression for predicting total bilirubin levels (all *p* < 0.001). The DeepSets model exhibited the highest R^2^ value at 0.782, indicating that it had a superior predictive performance among all the models tested. The Random Forest model had the second-highest R^2^ value of 0.708 and also showed the lowest mean difference (Figure 3). In contrast, despite its advanced deep learning architecture, the ResNet-50 model recorded the lowest R^2^ value at 0.506. ResNet-18 outperformed ResNet-50, achieving an R^2^ value of 0.712, yet it still fell short of surpassing traditional machine learning models, such as Random Forest (R^2^ = 0.738) and XGBoost (R^2^ = 0.708).

### 3.2. Confusion Matrix by Threshold

**Figure 4:**
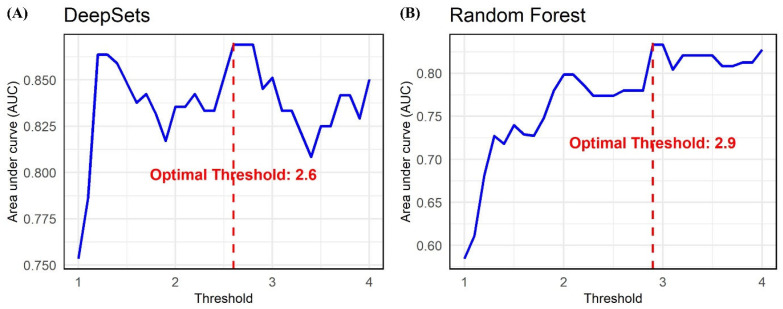
The optimal area under curve values when predicting jaundice using scleral images by varying the total bilirubin threshold from 1 to 4 in increments of 0.1 (left: DeepSets; right: Random Forest).

We selected DeepSets and Random Forest, the two algorithms that demonstrated the highest performance in regression, to further analyze their efficacy in predicting jaundice. By adjusting the threshold for jaundice prediction, we aimed to identify the optimal threshold that would yield the highest AUC. Using this optimal threshold, we then calculated the diagnostic accuracy of the models. The DeepSets model achieved the highest accuracy, with an AUC of 0.869, when the threshold was set at greater than or less than 2.6. Conversely, the Random Forest model exhibited an AUC of 0.833 with the threshold set at 2.9 (Figure 4).

When setting the thresholds at 2.6 and 2.9, the DeepSets model achieved an accuracy of 0.871, while the Random Forest model recorded an accuracy of 0.842 (Table 3). To assess the accuracy differences between the two models, we applied the respective thresholds and conducted the McNemar test. The test results showed no statistically significant differences in performance between the algorithms at either threshold, with *p*-values of 0.125 for 2.6 and 1.00 for 2.9.

## 4. Discussion

Various liver diseases, including impairments in hepatic metabolism or bilirubin transport or injury to any part of the hepatobiliary system, may result in hyperbilirubinemia [18]. In hepatobiliary patients, the early detection of jaundice enables timely intervention and helps prevent potential liver failure. Therefore, numerous studies have been conducted to develop a non-invasive, easy-to-use method for the early detection of jaundice. A notable example of such a method is transcutaneous bilirubinometry, which is commonly used in newborns [19]. This non-invasive technique measures skin reflectance at specific wavelengths of light to assess the extent of skin yellowing, thereby determining the severity of neonatal jaundice. Its simplicity has made it a popular choice in neonatal care units. Recently, the widespread availability of smartphones and advances in image processing have prompted efforts to analyze changes in scleral or skin color due to hyperbilirubinemia in adults, where traditional transcutaneous bilirubinometry is not applicable.

For the utilization of scleral images, the latest deep learning technologies could autonomously identify scleral regions if extensive datasets were available. However, with limited patient data, it becomes necessary to segment the scleral area and analyze the color values from this specific region. Essential steps in this process include white balance or color adjustment, as varying lighting conditions during image capture can significantly affect the color temperature. Part et al. [9] employed a patch made from a sheet of white paper with a rectangular hole, while Mariakakis et al. [20] used paper glasses when taking a facial photo. Our study adopted a simpler method, using only an A4 sheet of white paper for color correction, which facilitated the effective preprocessing of the scleral images. Some studies have developed and utilized equipment such as goggles to control light for research purposes [7,21], whereas others have relied solely on images without incorporating any additional equipment [6,22,23,24].

Jaundice prediction using images involves two primary tasks: predicting the exact bilirubin levels through regression and determining the presence or absence of jaundice, measured by accuracy. In their BiliScreen research, Mariakakis et al. [20] reported a Pearson correlation coefficient of 0.78 when using a smartphone and goggles. When using a box designed to block out ambient light, the correlation coefficient increased to 0.89. This underscores the importance of a controlled environment for accurate predictions. Similarly, our approach involved capturing images in an open setting, achieving a correlation coefficient of 0.79, which is consistent with previous studies. Prajapati et al. [22] achieved a correlation coefficient of 0.96 using their jScan smartphone application, demonstrating a high level of accuracy in its predictions.

For diagnosing jaundice, various detailed analysis methods and equipment have been used, with most approaches that utilize scleral images reporting around 90% accuracy [6,7,20,21,22,24]. In our study, the DeepSets algorithm, which yielded the best results, achieved an accuracy of 0.871. Considering that most studies were conducted with limited datasets of fewer than 100 subjects and used different criteria, it is more important to focus on the potential of scleral image-based analysis rather than on performance differences. Interestingly, after developing an algorithm to predict bilirubin levels, we used the predicted values to determine the optimal threshold for distinguishing between normal and jaundiced cases by varying the threshold from 1.5 to 4.0 in 0.1 increments. Using the two algorithms that showed the highest correlation with actual bilirubin levels, DeepSets, and Random Forest, the optimal thresholds were identified as 2.6 and 2.9, respectively. Scleral icterus is detectable when total serum bilirubin levels exceed 3 mg/dL [10]. Our study supports this well-established theory and further suggests the potential for the early detection of mild hyperbilirubinemia in the 2 to 3 mg/dL range. This level may not be easily noticeable but is higher than the normal range. This highlights the possible clinical utility of our approach for early diagnosis.

In this study, we not only utilized scleral images but also incorporated urine color analysis. This approach, though not previously attempted, was deemed viable due to the established correlation between jaundice and urine color [25]. Urine images were captured against a white background under consistent lighting conditions, eliminating the need for white balancing. However, the accuracy of predictions based on urine images did not match those made using only scleral images. Even when combined with scleral images, the results were still less accurate than those obtained from scleral images alone.

The limitations of this study include its reliance on a limited number of scleral images from both patients and healthy individuals, which restricts the analysis and prevents it from reflecting the real-world distribution of cases. Additionally, the clinical utility of this predictive model cannot be confirmed. To implement this model in clinical practice, a more extensive dataset and images captured in diverse environments will be necessary to minimize potential errors and improve its robustness.

Nevertheless, we demonstrate the potential for predicting bilirubin levels using scleral images, emphasizing the utility of a simple tool such as A4 white paper for color correction. Although the results of urine-based predictions were less effective, incorporating urine analysis into this study adds significant value.

## 5. Conclusions

The significance of our study lies in demonstrating the potential for jaundice screening using a non-invasive approach. However, further research is needed to determine whether this method can contribute to the early detection of clinical diseases.

## Figures and Tables

**Table 1:** Demographics.

	Cases (n = 57)	Controls (n = 31)	*p*
Age	50.2 ± 11.0	52.3 ± 15.1	0.501 *
Sex (M/F)	34/23	16/15	0.616 ^†^
Laboratory findings [median (IQR)]			
Total bilirubin	5.46 (4.18–13.31)	0.85 (0.63–1.11)	<0.001 ^‡^
Direct bilirubin	2.75 (1.87–6.07)	0.21 (0.14–0.36)	<0.001 ^‡^
AST	110 (72–234)	39 (24–76)	<0.001 ^‡^
ALT	35 (20–101)	41 (20–86)	0.701 ^‡^
ALP	136 (99–199)	83 (71–140)	0.003 ^‡^
**Etiology [*N*, %]**			
Alcoholic liver disease	32 (56.1%)		
HBV	15 (26.3%)		
HCV	1 (1.8%)		
Others	9 (15.8%)		

* *t*-test; ^†^ chi-square test; ^‡^ Wilcoxon rank-sum test.

**Table 2:** The comparison of regression results across algorithms using scleral images.

Algorithm	B (Std. Error)	R^2^	Mean Difference (SD)	*p*
DeepSets	1.092 (0.071)	0.782	0.43 (± 3.46)	<0.001
ResNet-18	1.446 (0.112)	0.712	0.35 (± 4.36)	<0.001
ResNet-50	1.509 (0.182)	0.506	1.48 (± 5.43)	<0.001
Random Forest	1.055 (0.077)	0.738	−0.02 (± 3.76)	<0.001
XGBoost	0.970 (0.076)	0.708	0.07 (± 3.96)	<0.001
Decision Tree	0.815 (0.069)	0.677	−0.18 (± 4.32)	<0.001

**Table 3:** The confusion matrix for jaundice prediction based on the thresholds that showed the highest AUC for both algorithms.

Algorithm	Threshold	Accuracy	Precision	Recall	F1 Score
DeepSets	2.6	0.871	0.902	0.881	0.891
Random Forest	2.9	0.842	0.860	0.881	0.871

## Data Availability

Data is unavailable because informed consent for sharing patient personal information was not obtained.

## Supplementary Materials

The following supporting information can be downloaded at: https://www.mdpi.com/article/10.3390/jcm14093125/s1, Text S1. Model Input Preprocessing; Text S2. Hyperparameters and training settings for algorithms; Figure S1. Example of Preprocessed Scleral Image and Corresponding Bilirubin Prediction; Table S1. Transparent Reporting of an artificial intelligent powered multivariable prediction model for Individual Prognosis Or Diagnosis (TRIPOD+AI) Checklist for prediction model development and validation; Table S2. Comparison of regression results across algorithm using urine images and a combination of both sclera and urine images.
