# Supplementary material for: Non-Invasive Jaundice Screening Using AI: Machine Learning Analysis of Sclera and Urine Images
Source: J Clin Med. 2025 Apr 30;14(9):3125. doi: 10.3390/jcm14093125 (PMC12072804; doi:10.3390/jcm14093125)
Supplement: Supplementary file 1 [file jcm-14-03125-s001.zip › jcm-3571425-supplementary.pdf]

Text S1. Model Input Preprocessing

All images used in this study were organized into paired sets consisting of the image itself, corresponding segmentation masks, and ground-truth bilirubin values.

1. Convolutional Neural Network-Based Models (ResNet-18, ResNet-50)

Since the input size for ResNet architectures is fixed at 224 × 224, preprocessing included the following steps:

- White balancing
- Resizing to 224 × 224
- Masking of regions outside the sclera or urine regions of interest (RoI) by setting them to zero. This was applied for fair comparison and to prevent overfitting, although it did not significantly affect performance.
- Converting RGB to YCbCr color space
- **Input dimension**: 224 × 224 × 3 (color space channels)

2. DeepSets

DeepSets does not accept images directly but instead takes an array of N data points. In our model, we used pixel values from the segmented RoI (sclera or urine) as input, effectively concatenating each 3-dimensional YCbCr pixel vector into an input set. Formally, this can be represented as:

$$f: \{\mathrm{x}_1,\ \mathrm{x}_2,\ \ldots \mathrm{x}_\mathrm{n} \text{ for } \mathrm{i} = 1 \text{ to } \mathrm{n}\} \rightarrow \mathrm{R}$$

- **Input dimension**: N × 3 (color space channels, where N is the number of pixels in the RoI)

3. Conventional Machine Learning Models

The models accept input in the form of one-dimensional feature vectors. Based on previous studies, we extracted the following features:

- Mean and median values of each channel in RGB, YCrCb, HSV, and Lab color spaces (24 dimensions) [1,2]
- Statistical features including variance, kurtosis, and skewness across RGB, YCrCb, HSV, and Lab spaces (3 dimensions) [3]
- Histogram distributions of each channel in RGB, YCrCb, and HSV color spaces (512 dimensions each; total of 512 × 3 = 1,536 dimensions)
- Dominant RGB values (3 dimensions) [4]
- Yellow index (1 dimension) [4]
- **Input dimension**: 24 + 3 + 1,536 + 3 + 1 = 1,567

References

1. de Greef L, Goel M, Seo MJ, et al. BiliCam: using mobile phones to monitor newborn jaundice. *Proceedings of the 2014 ACM International Joint Conference on Pervasive and Ubiquitous Computing*; 2014. ACM, New York, NY, USA: 331–342. [DOI: 10.1145/2632048.2632076]
2. Mariakakis A, Banks MA, Phillipi L, et al. BiliScreen: smartphone-based scleral jaundice monitoring for liver and pancreatic disorders. *Proc ACM Interact Mob Wearable Ubiquitous Technol* 2017; **1**(2): 20. [DOI: 10.1145/3090085]
3. Miah MMM, Tazim RJ, Johora FT, et al. Non-invasive bilirubin level quantification and jaundice detection by sclera image processing. *Proceedings of the 2019 IEEE Global Humanitarian Technology Conference (GHTC)*; 2019. IEEE: 1–7. [DOI: 10.1109/GHTC46095.2019.9033059]
4. Prajapati J, Das D, Udutalapally V, Mahapatra R, et al. jScan: Smartphone-assisted bilirubin quantification and jaundice screening. *IEEE Sensors J* 2023; **23**(21): 26654–26661. [DOI: 10.1109/JSEN.2023.3315452]

Text S2. Hyperparameters and training settings for algorithms

We conducted experiments for six different models: DeepSets, ResNet-18, ResNet-50, Random Forest, Decision Tree, and XGBoost [1-5]. All models were trained and evaluated using stratified 5-fold cross-validation. Hyperparameter settings and training procedures were tailored for optimal performance. The DeepSets model was implemented following the original architecture, with minor modifications. Specifically, we utilized a 7-layer Multilayer Perceptron (MLP) with 256 channels for ϕ and a 3-layer MLP with 128 channels for ρ. We employed Exponential Linear Units (ELU) as the activation function for ϕ, while the Tanh activation function was used for ρ . For the ResNet-18 and ResNet-50 models, we followed the original implementations without any architectural modifications. Both models were trained using pretrained weights from ImageNet to ensure stable training under the small dataset. [6] Random Forest, Decision Tree and XGBoost were implemented using the standard configurations, as provided by scikit-learn and XGBoost. Although hyperparameter tuning was performed, the standard configurations provided by scikit-learn and XGBoost yielded the best results showing no meaningful improvement over the tuned versions.

All neural network models were trained for 5,000 iterations. To ensure stable convergence, we applied a learning rate scheduling strategy, where the learning rate was reduced by 10% after every 200 iterations. The models were trained using Stochastic Gradient Descent (SGD), which has proven effective for a wide range of machine learning tasks. Same feature extraction and preprocessing pipeline used for sclera images was applied to urine images. For the ensemble approach, DeepSets and ResNet concatenated the feature vectors extracted by two independent feature extractors (ϕ for DeepSets) to combine sclera and urine data. Traditional models concatenated the feature vectors derived from the extraction process. This unified representation was then used as input for the final prediction.

References

1 Zaheer M, Kottur S, Ravanbakhsh S, Poczos B, Salakhutdinov RR, Smola AJ. Deep sets. *Advances in neural information processing systems* 2017; **30**

2 He K, Zhang X, Ren S, Sun J. Deep residual learning for image recognition. Proceedings of the Proceedings of the IEEE conference on computer vision and pattern recognition; 2016. 770-778

3 Song Y-Y, Ying L. Decision tree methods: applications for classification and prediction. *Shanghai archives of psychiatry* 2015; **27**(2): 130

4 Breiman L. Random Forests. *Machine Learning* 2001; **45**(1): 5-32 [DOI: 10.1023/A:1010933404324]

5 Chen T, Guestrin C. Xgboost: A scalable tree boosting system. Proceedings of the Proceedings of the 22nd acm sigkdd international conference on knowledge discovery and data mining; 2016. 785-794

6 Neyshabur B, Sedghi H, Zhang C. What is being transferred in transfer learning? *Advances in neural information processing systems* 2020; **33**: 512-523

Figure S1. Example of Preprocessed Scleral Image and Corresponding Bilirubin Prediction

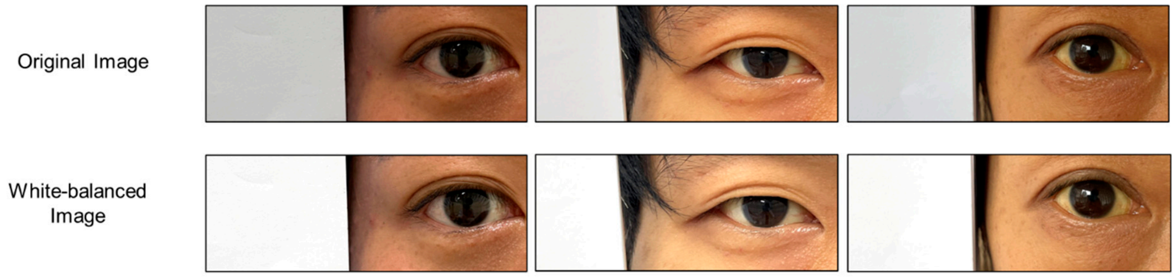

Bilirubin level

**Ground truth**	1.60	4.63	13.82
**Prediction**			
DeepSets	1.98	4.64	13.41
ResNet-50	3.35	5.51	9.22
Random Forest	1.83	4.41	12.46

Table S1. Transparent Reporting of an artificial intelligent powered multivariable prediction model for Individual Prognosis Or Diagnosis (TRIPOD+AI) Checklist for prediction model development and validation.

Section/Topic	Item		Checklist Item	Page
**Title**				
Title	1	D;E	Identify the study as developing or evaluating the performance of a multivariable prediction model, the target population, and the outcome to be predicted	1
**Abstract**				
Abstract	2	D;E	See TRIPOD+AI for Abstracts checklist	Table S1. TRIPOD+AI for Abstracts checklist
**Introduction**				
Background	3a	D;E	Explain the healthcare context (including whether diagnostic or prognostic) and rationale for developing or evaluating the prediction model, including references to existing models	1,2
	3b	D;E	Describe the target population and the intended purpose of the prediction model in the context of the care pathway, including its intended users (eg, healthcare professionals, patients, public)	1,2,3
	3c	D;E	Describe any known health inequalities between sociodemographic groups	N/A
Objectives	4	D;E	Specify the study objectives, including whether the study describes the development or validation of a prediction model (or both)	2
**Methods**				
Data	5a	D;E	Describe the sources of data separately for the development and evaluation datasets (eg, randomised trial, cohort, routine care or registry data), the rationale for using these data, and representativeness of the data	2,3
	5b	D;E	Specify the dates of the collected participant data, including start and end of participant accrual; and, if applicable, end of follow-up	2
Participants	6a	D;E	Specify key elements of the study setting (eg, primary care, secondary care, general population) including the number and location of centres	2,3
	6b	D;E	Describe the eligibility criteria for study participants	2,3
	6c	D;E	Give details of any treatments received, and how they were handled during model development or evaluation, if relevant	N/A
Data preparation	7	D;E	Describe any data pre-processing and quality checking, including whether this was similar across relevant sociodemographic groups	3,4
Outcome	8a	D;E	Clearly define the outcome that is being predicted and the time horizon, including how and when assessed, the rationale for choosing this outcome, and whether the method of outcome assessment is consistent across sociodemographic groups	3
	8b	D;E	If outcome assessment requires subjective interpretation, describe the qualifications and demographic characteristics of the outcome assessors	N/A
	8c	D;E	Report any actions to blind assessment of the outcome to be predicted	N/A
Predictors	9a	D	Describe the choice of initial predictors (eg, literature, previous models, all available predictors) and any pre-selection of predictors before model building	3,4

	9b	D;E	Clearly define all predictors, including how and when they were measured (and any actions to blind assessment of predictors for the outcome and other predictors)	3,4
	9c	D;E	If predictor measurement requires subjective interpretation, describe the qualifications and demographic characteristics of the predictor assessors	N/A
Sample size	10	D;E	Explain how the study size was arrived at (separately for development and evaluation), and justify that the study size was sufficient to answer the research question. Include details of any sample size calculation	3
Missing data	11	D;E	Describe how missing data were handled. Provide reasons for omitting any data	No missing data
Analytical methods	12a	D	Describe how the data were used (eg, for development and evaluation of model performance) in the analysis, including whether the data were partitioned, considering any sample size requirements	4,5
	12b	D	Depending on the type of model, describe how predictors were handled in the analyses (functional form, rescaling, transformation, or any standardisation)	4,5, Supplementary Text S1
	12c	D	Specify the type of model, rationale†, all model building steps, including any hyperparameter tuning, and method for internal validation	4,5, Supplementary Text S1
	12d	D;E	Describe if and how any heterogeneity in estimates of model parameter values and model performance was handled and quantified across clusters (eg, hospitals, countries). See TRIPOD-Cluster for additional considerations‡	Supplementary Text S1
	12e	D;E	Specify all measures and plots used (and their rationale) to evaluate model performance (eg, discrimination, calibration, clinical utility) and, if relevant, to compare multiple models	4,5
	12f	E	Describe any model updating (eg, recalibration) arising from the model evaluation, either overall or for particular sociodemographic groups or settings	N/A
	12g	E	For model evaluation, describe how the model predictions were calculated (eg, formula, code, object, application programming interface)	5, Supplementary Text S1
Class imbalance	13	D;E	If class imbalance methods were used, state why and how this was done, and any subsequent methods to recalibrate the model or the model predictions	N/A
Fairness	14	D;E	Describe any approaches that were used to address model fairness and their rationale	3, Supplementary Text S1
Model output	15	D	Specify the output of the prediction model (eg, probabilities, classification). Provide details and rationale for any classification and how the thresholds were identified	3,4
Training versus evaluation	16	D;E	Identify any differences between the development and evaluation data in healthcare setting, eligibility criteria, outcome, and predictors	4
Ethical approval	17	D;E	Name the institutional research board or ethics committee that approved the study and describe the participant informed consent or the ethics committee waiver of informed consent	2
**Open science**				
Funding	18a	D;E	Give the source of funding and the role of the funders for the present study	10
Conflicts of interest	18b	D;E	Declare any conflicts of interest and financial disclosures for all authors	10
Protocol	18c	D;E	Indicate where the study protocol can be accessed or state that a protocol was not prepared	10
Registration	18d	D;E	Provide registration information for the study, including register name and registration number, or state that the study was not registered	10
Data sharing	18e	D;E	Provide details of the availability of the study data	10
Code sharing	18f	D;E	Provide details of the availability of the analytical code§	N/A

**Patient and public involvement**				
Patient and public involvement	19	D;E	Provide details of any patient and public involvement during the design, conduct, reporting, interpretation, or dissemination of the study or state no involvement	N/A
**Results**				
Participants	20a	D;E	Describe the flow of participants through the study, including the number of participants with and without the outcome and, if applicable, a summary of the follow-up time. A diagram may be helpful	5
	20b	D;E	Report the characteristics overall and, where applicable, for each data source or setting, including the key dates, key predictors (including demographics), treatments received, sample size, number of outcome events, follow-up time, and amount of missing data. A table may be helpful. Report any differences across key demographic groups	5, Table 1
	20c	E	For model evaluation, show a comparison with the development data of the distribution of important predictors (demographics, predictors, and outcome)	5, Table 1
Model development	21	D;E	Specify the number of participants and outcome events in each analysis (eg, for model development, hyperparameter tuning, model evaluation)	N/A
Model specification	22	D	Provide details of the full prediction model (eg, formula, code, object, application programming interface) to allow predictions in new individuals and to enable third party evaluation and implementation, including any restrictions to access or reuse (eg, freely available, proprietary)¶	N/A
Model performance	23a	D;E	Report model performance estimates with confidence intervals, including for any key subgroups (eg, sociodemographic). Consider plots to aid presentation	Tables 2 and 3, Figures 3 and 4
	23b	D;E	If examined, report results of any heterogeneity in model performance across clusters. See TRIPOD-Cluster for additional details‡	N/A
Model updating	24	E	Report the results from any model updating, including the updated model and subsequent performance	N/A
**Discussion**				
Interpretation	25	D;E	Give an overall interpretation of the main results, including issues of fairness in the context of the objectives and previous studies	7,8,9
Limitations	26	D;E	Discuss any limitations of the study (such as a non-representative sample, sample size, overfitting, missing data) and their effects on any biases, statistical uncertainty, and generalisability	7,8,9
Usability of the model in the context of current care	27a	D	Describe how poor quality or unavailable input data (eg, predictor values) should be assessed and handled when implementing the prediction model	N/A
	27b	D	Specify whether users will be required to interact in the handling of the input data or use of the model, and what level of expertise is required of users	N/A
	27c	D;E	Discuss any next steps for future research, with a specific view to applicability and generalisability of the model	7,8,9

* D=items relevant only to the development of a prediction model; E=items relating solely to the evaluation of a prediction model; D;E=items applicable to both the development and evaluation of a prediction model.

† Separately for all model building approaches.

‡ TRIPOD-Cluster is a checklist of reporting recommendations for studies developing or validating models that explicitly account for clustering or explore heterogeneity in model performance (eg, at different hospitals or centres).

§ Relates to the analysis code, for example, any data cleaning, feature engineering, model building, and evaluation.

¶ Relates to the code to implement the model to get estimates of risk for a new individual.

TRIPOD+AI for Abstracts checklist		
Section and item	Checklist item	Included in Abstract
**Title**		
1	Identify the study as developing or evaluating the performance of a multivariable prediction model, the target population, and the outcome to be predicted	**YES**
**Background**		
2	Provide a brief explanation of the healthcare context and rationale for developing or evaluating the performance of all models	**YES**
**Objectives**		
3	Specify the study objectives, including whether the study describes model development, evaluation, or both	**YES**
**Methods**		
4	Describe the sources of data	**YES**
5	Describe the eligibility criteria and setting where the data were collected	**YES**
6	Specify the outcome to be predicted by the model, including time horizon of predictions in case of prognostic models	**YES**
7	Specify the type of model, a summary of the model-building steps, and the method for internal validation†	**YES**
8	Specify the measures used to assess model performance (eg, discrimination, calibration, clinical utility)	**YES**
**Results**		
9	Report the number of participants and outcome events	**YES**
10	Summarise the predictors in the final model†	**YES**
11	Report model performance estimates (with confidence intervals)	**YES**
**Discussion**		
12	Give an overall interpretation of the main results	**YES**
**Registration**		
13	Give the registration number and name of the registry or repository	**YES**

† Relevant only to studies describing the development of a prediction model.

Table S2. Comparison of regression results across algorithm using urine images and a combination of both sclera and urine images.

**Algorithm**	**Source**	**B (Std. error)**	**R^2**	**Mean difference (SD)**	***p***
DeepSets	Urine	1.381 (0.357)	0.179	2.20 (± 6.68)	<.001
ResNet-18	Urine	0.786 (0.454)	0.041	-0.96 (± 7.17)	0.088
ResNet-50	Urine	5.115 (0.177)	0.108	4.98 (± 7.17)	0.005
Random Forest	Urine	0.681 (0.172)	0.185	-0.11 (± 6.76)	<.001
XGBoost	Urine	0.465 (0.129)	0.159	-0.57 (± 7.49)	<.001
Decision Tree	Urine	0.320 (0.110)	0.108	-0.55 (± 8.58)	0.005
DeepSets	Sclera + Urine	1.047 (0.082)	0.704	0.47 (± 3.98)	<.001
ResNet-18	Sclera + Urine	1.547 (0.170)	0.545	-0.34 (± 5.28)	<.001
ResNet-50	Sclera + Urine	2.319 (0.337)	0.407	3.38 (± 6.22)	<.001
Random Forest	Sclera + Urine	1.061 (0.079)	0.723	-0.04 (± 3.86)	<.001
XGBoost	Sclera + Urine	0.979 (0.070)	0.738	-0.27 (± 3.74)	<.001
Decision Tree	Sclera + Urine	0.845 (0.073)	0.663	0.54 (± 4.38)	<.001
